# 3,10-*C*-*meso*-3,5,7,7,10,12,14,14-Octa­methyl-4,11-diaza-1,8-diazo­niacyclo­tetra­decane bis­(perchlorate)

**DOI:** 10.1107/S1600536810030217

**Published:** 2010-08-04

**Authors:** Tapashi G. Roy, Saroj K. S. Hazari, Kanak K. Barua, Provi Palit, Edward R. T. Tiekink

**Affiliations:** a Department of Chemistry, University of Chittagong, Chittagong 4331, Bangladesh; b Department of Chemistry, University of Malaya, 50603 Kuala Lumpur, Malaysia

## Abstract

The structure determination of the title salt, C_18_H_42_N_4_^2+^·2ClO_4_^−^, reveals that protonation has occurred at diagonally opposite amine N atoms. Intra­molecular N—H⋯N hydrogen bonds stabilize the conformation of the dication. In the crystal, the dications are bridged by perchlorate ions *via* N—H⋯O hydrogen bonds into supra­molecular chains propagating along the *c* axis and weak C—H⋯O inter­actions cross-link the chains.

## Related literature

For background to macrocycles and for related structures, see: Benson *et al.* (2006 ▶); Roy *et al.* (2006 ▶, 2008 ▶); Hazari *et al.* (2008 ▶). For the synthesis, see: Curtis *et al.* (1969 ▶); Bembi *et al.* (1989 ▶).
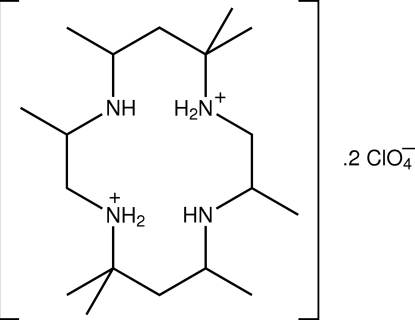


## Experimental

### 

#### Crystal data

C_18_H_42_N_4_^2+^·2ClO_4_^−^
*M* *_r_* = 513.46
Monoclinic, 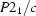

*a* = 8.868 (2) Å
*b* = 16.297 (3) Å
*c* = 17.754 (5) Å
β = 102.088 (5)°
*V* = 2508.9 (10) Å^3^
*Z* = 4
Mo *K*α radiation
μ = 0.31 mm^−1^
*T* = 98 K
0.35 × 0.10 × 0.03 mm

#### Data collection

Rigaku AFC12/SATURN724 diffractometer
Absorption correction: multi-scan (*ABSCOR*; Higashi, 1995 ▶) *T*_min_ = 0.695, *T*_max_ = 1
54635 measured reflections
4387 independent reflections
4279 reflections with *I* > 2σ(*I*)
*R*_int_ = 0.055

#### Refinement

*R*[*F* ^2^ > 2σ(*F* ^2^)] = 0.078
*wR*(*F* ^2^) = 0.240
*S* = 1.19
4387 reflections
295 parameters
2 restraints
H-atom parameters constrained
Δρ_max_ = 0.85 e Å^−3^
Δρ_min_ = −0.73 e Å^−3^

### 

Data collection: *CrystalClear* (Molecular Structure Corporation & Rigaku, 2005 ▶); cell refinement: *CrystalClear*; data reduction: *CrystalClear*; program(s) used to solve structure: *SHELXS97* (Sheldrick, 2008 ▶); program(s) used to refine structure: *SHELXL97* (Sheldrick, 2008 ▶); molecular graphics: *ORTEPII* (Johnson, 1976 ▶) and *DIAMOND* (Brandenburg, 2006 ▶); software used to prepare material for publication: *publCIF* (Westrip, 2010 ▶).

## Supplementary Material

Crystal structure: contains datablocks global, I. DOI: 10.1107/S1600536810030217/hb5587sup1.cif

Structure factors: contains datablocks I. DOI: 10.1107/S1600536810030217/hb5587Isup2.hkl

Additional supplementary materials:  crystallographic information; 3D view; checkCIF report

## Figures and Tables

**Table 1:** Hydrogen-bond geometry (Å, °)

*D*—H⋯*A*	*D*—H	H⋯*A*	*D*⋯*A*	*D*—H⋯*A*
N1—H2n⋯N11	0.92	2.07	2.841 (4)	140
N8—H9n⋯N4	0.92	1.95	2.763 (5)	146
N1—H1n⋯O2	0.92	2.37	2.963 (4)	122
N1—H1n⋯O7^i^	0.92	2.41	3.169 (4)	140
N1—H2n⋯N4	0.92	2.56	2.929 (4)	105
N4—H4n⋯O6	0.88	2.38	3.212 (4)	158
N8—H8n⋯O1	0.92	2.02	2.937 (5)	177
N11—H11n⋯O6	0.88	2.24	3.087 (5)	162
C2—H2a⋯O1^ii^	0.99	2.57	3.109 (5)	114
C9—H9a⋯O5^iii^	0.99	2.57	3.498 (6)	155
C12a—H12b⋯O8	0.98	2.56	3.487 (5)	158

Symmetry codes: (i) 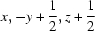; (ii) 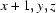; (iii) 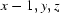.

**Fig. 1.**
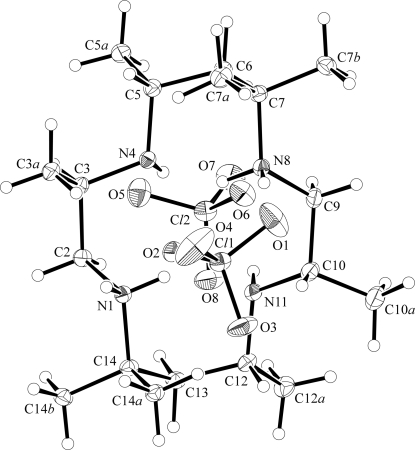
The molecular structure of (I) showing displacement ellipsoids at the 50% probability level.

**Fig. 2.**
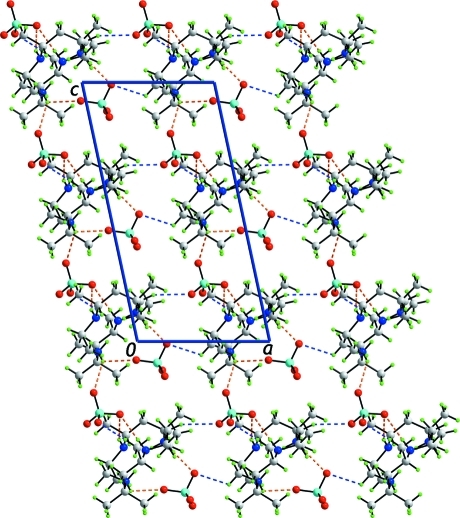
Supramolecular array in (I) showing N–H···O hydrogen bonds and C–H···O contacts as orange and blue dashed lines, respectively. Colour code: Cl (cyan), O (red), N (blue), C (grey) & H (green).

**Fig. 3.**
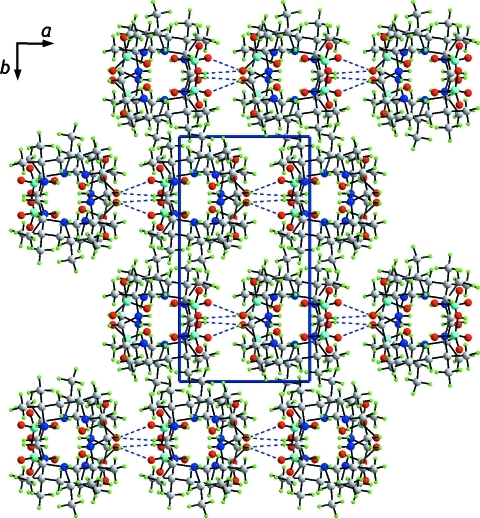
Stacking of layers along the b axis in (I). The C–H···O contacts are shown as blue dashed lines. Colour code: Cl (cyan), O (red), N (blue), C (grey) & H (green).

## supplementary crystallographic information

### Comment

The title salt (I) was characterized during studies of macrocyclic ligands of this type as well as their transition metal complexes (Benson *et al.*, 2006; Roy *et al.*, 2006; Hazari *et al.*, 2008; Roy *et al.*, 2008). The asymmetric unit of (I) comprises one di-protonated macrocyclic ligand and two perchlorate anions, Fig. 1. The observed conformation and pattern of protonation (*i.e*. at the diagonally opposite N1 and N8 atoms) in the di-cation conforms to expectation (Hazari *et al.*, 2008). The presence of N–H···N hydrogen bonds within the cavity is noted, Table 1. The remaining N–H groups form N–H···O hydrogen bonds to perchlorate-O atoms, Table 1. The perchlorate ions serve to bridge the cations to form a supramolecular chain along the *c* axis and connections between these, *i.e. via* C–H···O contacts, lead to the formation of a supramolecular layer in the *ac* plane. Layers stack along the *b* axis, Fig. 3.

### Experimental

The compound 3,10-*C*-*meso*-3,5,7,7,10,12,14,14—octamethyl-4,11-diaza-1,8-diazoniacyclotetradecadiene (Curtis *et al.*, 1969), on reduction with NaBH_4_, yields an isomeric mixture of saturated macrocycles, the Me_8_[14] anes, which have been resolved into three distinct isomers (Bembi *et al.*, 1989). During synthesis of Fe(III) complex of one of the isomers, *L*_C_ (Bembi *et al.*, 1989), this isomeric ligand on heating with slightly acidic methanolic solution of Fe_2_(ClO_4_)_3_.6H_2_O in the ratio of 1:2, followed by cooling and slow evaporation at room temperature for a few days produced yellow-orange prisms of (I).

### Refinement

All N– and C-bound H atoms were allowed to ride on their parent atoms at N–H and C–H distances of 0.88–0.92 Å and 0.92–1.00 Å, respectively, and with *U*_iso_(H) values of 1.2–1.5*U*_eq_(parent atom).

### Crystal data

**Table:**

C_18_H_42_N_4_^2+^·2ClO_4_^−^	*F*(000) = 1104
*M**_r_* = 513.46	*D*_x_ = 1.359 Mg m^−^^3^
Monoclinic, *P*2_1_/*c*	Mo *K*α radiation, λ = 0.71073 Å
Hall symbol: -P 2ybc	Cell parameters from 7813 reflections
*a* = 8.868 (2) Å	θ = 2.4–30.4°
*b* = 16.297 (3) Å	µ = 0.31 mm^−^^1^
*c* = 17.754 (5) Å	*T* = 98 K
β = 102.088 (5)°	Prism, yellow-orange
*V* = 2508.9 (10) Å^3^	0.35 × 0.10 × 0.03 mm
*Z* = 4	

### Data collection

**Table:**

Rigaku AFC12K/SATURN724 diffractometer	4387 independent reflections
Radiation source: fine-focus sealed tube	4279 reflections with *I* > 2σ(*I*)
graphite	*R*_int_ = 0.055
ω scans	θ_max_ = 25.0°, θ_min_ = 2.4°
Absorption correction: multi-scan (*ABSCOR*; Higashi, 1995)	*h* = −10→10
*T*_min_ = 0.695, *T*_max_ = 1	*k* = −19→19
54635 measured reflections	*l* = −21→21

### Refinement

**Table:**

Refinement on *F*^2^	Primary atom site location: structure-invariant direct methods
Least-squares matrix: full	Secondary atom site location: difference Fourier map
*R*[*F*^2^ > 2σ(*F*^2^)] = 0.078	Hydrogen site location: inferred from neighbouring sites
*wR*(*F*^2^) = 0.240	H-atom parameters constrained
*S* = 1.19	*w* = 1/[σ^2^(*F*_o_^2^) + (0.128*P*)^2^ + 4.0207*P*] where *P* = (*F*_o_^2^ + 2*F*_c_^2^)/3
4387 reflections	(Δ/σ)_max_ = 0.001
295 parameters	Δρ_max_ = 0.85 e Å^−^^3^
2 restraints	Δρ_min_ = −0.73 e Å^−^^3^

### Special details

**Table:**

Geometry. All e.s.d.'s (except the e.s.d. in the dihedral angle between two l.s. planes) are estimated using the full covariance matrix. The cell e.s.d.'s are taken into account individually in the estimation of e.s.d.'s in distances, angles and torsion angles; correlations between e.s.d.'s in cell parameters are only used when they are defined by crystal symmetry. An approximate (isotropic) treatment of cell e.s.d.'s is used for estimating e.s.d.'s involving l.s. planes.
Refinement. Refinement of *F*^2^ against ALL reflections. The weighted *R*-factor *wR* and goodness of fit *S* are based on *F*^2^, conventional *R*-factors *R* are based on *F*, with *F* set to zero for negative *F*^2^. The threshold expression of *F*^2^ > σ(*F*^2^) is used only for calculating *R*-factors(gt) *etc*. and is not relevant to the choice of reflections for refinement. *R*-factors based on *F*^2^ are statistically about twice as large as those based on *F*, and *R*- factors based on ALL data will be even larger.

### Fractional atomic coordinates and isotropic or equivalent isotropic displacement parameters (Å^2^)

**Table:**

	*x*	*y*	*z*	*U*_iso_*/*U*_eq_	
Cl1	−0.11642 (10)	0.18918 (6)	0.58280 (6)	0.0337 (3)	
Cl2	0.40297 (12)	0.32683 (6)	0.27709 (6)	0.0372 (3)	
O1	−0.2173 (4)	0.1783 (4)	0.5090 (2)	0.0900 (17)	
O2	0.0398 (3)	0.18064 (16)	0.57180 (16)	0.0351 (6)	
O3	−0.1414 (4)	0.2682 (2)	0.6113 (2)	0.0641 (11)	
O4	−0.1502 (6)	0.1294 (3)	0.6341 (3)	0.0955 (17)	
O5	0.5140 (4)	0.2692 (2)	0.3177 (2)	0.0593 (9)	
O6	0.2511 (4)	0.2976 (2)	0.28141 (18)	0.0506 (8)	
O7	0.4141 (4)	0.3316 (2)	0.19821 (18)	0.0551 (9)	
O8	0.4289 (4)	0.4058 (2)	0.3127 (2)	0.0608 (10)	
N1	0.3158 (3)	0.26608 (17)	0.54123 (17)	0.0251 (6)	
H1N	0.2939	0.2296	0.5770	0.030*	
H2N	0.2306	0.2683	0.5015	0.030*	
N4	0.2753 (3)	0.15534 (18)	0.40857 (16)	0.0253 (6)	
H4N	0.2973	0.1951	0.3791	0.030*	
N8	−0.0379 (4)	0.18381 (18)	0.38755 (18)	0.0280 (7)	
H8N	−0.0926	0.1801	0.4261	0.034*	
H9N	0.0646	0.1883	0.4110	0.034*	
N11	0.1144 (4)	0.35008 (19)	0.42023 (18)	0.0292 (7)	
H11N	0.1560	0.3470	0.3795	0.035*	
C2	0.4467 (4)	0.2319 (2)	0.5101 (2)	0.0287 (8)	
H2A	0.5392	0.2281	0.5523	0.034*	
H2B	0.4708	0.2698	0.4707	0.034*	
C3	0.4093 (4)	0.1473 (2)	0.4745 (2)	0.0276 (8)	
H3	0.5000	0.1284	0.4539	0.033*	
C3A	0.3788 (5)	0.0843 (2)	0.5324 (2)	0.0347 (9)	
H3A1	0.3546	0.0313	0.5066	0.052*	
H3A2	0.2915	0.1022	0.5542	0.052*	
H3A3	0.4706	0.0786	0.5738	0.052*	
C5	0.2380 (4)	0.0798 (2)	0.3613 (2)	0.0284 (8)	
H5	0.2272	0.0334	0.3966	0.034*	
C5A	0.3644 (4)	0.0571 (3)	0.3177 (2)	0.0352 (9)	
H5A1	0.4621	0.0487	0.3547	0.053*	
H5A2	0.3763	0.1016	0.2823	0.053*	
H5A3	0.3356	0.0064	0.2885	0.053*	
C6	0.0830 (4)	0.0920 (2)	0.3062 (2)	0.0298 (8)	
H6A	0.0640	0.0441	0.2713	0.036*	
H6B	0.0910	0.1409	0.2742	0.036*	
C7	−0.0589 (4)	0.1032 (2)	0.3428 (2)	0.0277 (8)	
C7A	−0.0691 (5)	0.0366 (2)	0.4014 (2)	0.0371 (9)	
H7A1	−0.1593	0.0465	0.4239	0.056*	
H7A2	0.0244	0.0374	0.4421	0.056*	
H7A3	−0.0790	−0.0170	0.3759	0.056*	
C7B	−0.2054 (4)	0.1054 (3)	0.2809 (2)	0.0359 (9)	
H7B1	−0.1974	0.1487	0.2436	0.054*	
H7B2	−0.2937	0.1165	0.3046	0.054*	
H7B3	−0.2199	0.0523	0.2544	0.054*	
C9	−0.0841 (5)	0.2612 (2)	0.3447 (2)	0.0359 (9)	
H9A	−0.1952	0.2585	0.3205	0.043*	
H9B	−0.0259	0.2667	0.3031	0.043*	
C10	−0.0545 (5)	0.3363 (2)	0.3968 (3)	0.0388 (10)	
H10	−0.0939	0.3240	0.4444	0.047*	
C10A	−0.1490 (5)	0.4068 (3)	0.3556 (4)	0.0603 (15)	
H10A	−0.1333	0.4557	0.3884	0.091*	
H10B	−0.2585	0.3919	0.3444	0.091*	
H10C	−0.1163	0.4184	0.3072	0.091*	
C12	0.1636 (4)	0.4273 (2)	0.4622 (2)	0.0303 (8)	
H12	0.0878	0.4406	0.4950	0.036*	
C12A	0.1744 (5)	0.5016 (2)	0.4101 (2)	0.0382 (9)	
H12A	0.0739	0.5111	0.3759	0.057*	
H12B	0.2516	0.4907	0.3792	0.057*	
H12C	0.2045	0.5503	0.4421	0.057*	
C13	0.3223 (4)	0.4157 (2)	0.5151 (2)	0.0307 (8)	
H13A	0.3547	0.4691	0.5398	0.037*	
H13B	0.3964	0.4017	0.4824	0.037*	
C14	0.3374 (4)	0.3508 (2)	0.5788 (2)	0.0283 (8)	
C14A	0.2108 (4)	0.3575 (2)	0.6251 (2)	0.0303 (8)	
H14A	0.1096	0.3539	0.5902	0.045*	
H14B	0.2198	0.4103	0.6522	0.045*	
H14C	0.2217	0.3127	0.6627	0.045*	
C14B	0.4963 (4)	0.3570 (2)	0.6320 (2)	0.0324 (8)	
H14D	0.5052	0.3157	0.6728	0.049*	
H14E	0.5095	0.4119	0.6550	0.049*	
H14F	0.5761	0.3474	0.6023	0.049*	

### Atomic displacement parameters (Å^2^)

**Table:**

	*U*^11^	*U*^22^	*U*^33^	*U*^12^	*U*^13^	*U*^23^
Cl1	0.0281 (5)	0.0333 (5)	0.0438 (6)	−0.0019 (3)	0.0170 (4)	−0.0056 (4)
Cl2	0.0403 (6)	0.0380 (6)	0.0367 (6)	−0.0025 (4)	0.0157 (4)	−0.0013 (4)
O1	0.0288 (18)	0.183 (5)	0.060 (2)	0.005 (2)	0.0121 (16)	−0.045 (3)
O2	0.0232 (13)	0.0397 (15)	0.0448 (16)	0.0018 (11)	0.0129 (12)	−0.0050 (12)
O3	0.050 (2)	0.0422 (19)	0.108 (3)	−0.0004 (15)	0.033 (2)	−0.0312 (19)
O4	0.105 (4)	0.058 (2)	0.156 (4)	0.017 (2)	0.099 (3)	0.041 (3)
O5	0.054 (2)	0.059 (2)	0.064 (2)	0.0037 (16)	0.0102 (17)	0.0140 (17)
O6	0.0411 (18)	0.065 (2)	0.0484 (18)	−0.0130 (15)	0.0167 (14)	−0.0025 (15)
O7	0.066 (2)	0.064 (2)	0.0416 (18)	−0.0099 (17)	0.0256 (16)	0.0037 (15)
O8	0.059 (2)	0.048 (2)	0.079 (2)	−0.0058 (16)	0.0246 (19)	−0.0237 (17)
N1	0.0215 (14)	0.0253 (15)	0.0294 (15)	−0.0008 (11)	0.0076 (12)	−0.0010 (12)
N4	0.0254 (15)	0.0241 (15)	0.0266 (15)	0.0005 (12)	0.0060 (12)	0.0015 (11)
N8	0.0244 (15)	0.0257 (16)	0.0331 (16)	0.0010 (11)	0.0038 (12)	−0.0012 (12)
N11	0.0270 (16)	0.0243 (15)	0.0351 (17)	0.0005 (12)	0.0034 (13)	−0.0022 (13)
C2	0.0209 (16)	0.0318 (19)	0.0351 (19)	−0.0021 (14)	0.0100 (14)	−0.0056 (15)
C3	0.0215 (17)	0.0288 (19)	0.0335 (19)	0.0040 (14)	0.0077 (14)	−0.0035 (15)
C3A	0.042 (2)	0.0269 (19)	0.0326 (19)	0.0072 (16)	0.0011 (16)	0.0004 (15)
C5	0.0285 (18)	0.0262 (18)	0.0306 (18)	0.0004 (14)	0.0066 (15)	0.0007 (14)
C5A	0.033 (2)	0.040 (2)	0.033 (2)	0.0050 (16)	0.0093 (16)	−0.0067 (16)
C6	0.0291 (19)	0.0305 (19)	0.0285 (18)	−0.0015 (15)	0.0031 (15)	−0.0012 (14)
C7	0.0265 (18)	0.0245 (18)	0.0322 (18)	−0.0002 (14)	0.0063 (15)	−0.0017 (14)
C7A	0.037 (2)	0.029 (2)	0.047 (2)	−0.0034 (16)	0.0130 (18)	0.0046 (17)
C7B	0.030 (2)	0.039 (2)	0.037 (2)	−0.0044 (16)	0.0045 (16)	−0.0062 (17)
C9	0.0295 (19)	0.0266 (19)	0.046 (2)	0.0020 (15)	−0.0040 (17)	0.0040 (16)
C10	0.029 (2)	0.028 (2)	0.057 (3)	0.0031 (15)	0.0028 (18)	−0.0041 (18)
C10A	0.038 (2)	0.036 (2)	0.098 (4)	0.0065 (19)	−0.009 (3)	−0.004 (2)
C12	0.0304 (19)	0.0231 (18)	0.039 (2)	0.0022 (14)	0.0106 (16)	0.0008 (15)
C12A	0.042 (2)	0.0270 (19)	0.045 (2)	−0.0008 (16)	0.0084 (18)	0.0078 (16)
C13	0.0320 (19)	0.0232 (18)	0.037 (2)	−0.0056 (14)	0.0078 (16)	−0.0025 (15)
C14	0.0271 (18)	0.0242 (18)	0.0342 (19)	−0.0027 (14)	0.0077 (15)	−0.0041 (14)
C14A	0.0298 (19)	0.0287 (18)	0.0336 (19)	0.0019 (14)	0.0095 (15)	−0.0044 (15)
C14B	0.0285 (19)	0.032 (2)	0.035 (2)	−0.0031 (15)	0.0044 (15)	−0.0068 (15)

### Geometric parameters (Å, °)

**Table:**

Cl1—O4	1.408 (4)	C6—C7	1.542 (5)
Cl1—O3	1.418 (3)	C6—H6A	0.9900
Cl1—O1	1.435 (4)	C6—H6B	0.9900
Cl1—O2	1.445 (3)	C7—C7B	1.516 (5)
Cl2—O7	1.427 (3)	C7—C7A	1.520 (5)
Cl2—O8	1.431 (3)	C7A—H7A1	0.9800
Cl2—O5	1.440 (4)	C7A—H7A2	0.9800
Cl2—O6	1.446 (3)	C7A—H7A3	0.9800
N1—C2	1.493 (4)	C7B—H7B1	0.9800
N1—C14	1.528 (4)	C7B—H7B2	0.9800
N1—H1N	0.9200	C7B—H7B3	0.9800
N1—H2N	0.9200	C9—C10	1.524 (6)
N4—C5	1.489 (4)	C9—H9A	0.9900
N4—C3	1.489 (4)	C9—H9B	0.9900
N4—H4N	0.8800	C10—C10A	1.517 (6)
N8—C9	1.485 (5)	C10—H10	1.0000
N8—C7	1.526 (4)	C10A—H10A	0.9800
N8—H8N	0.9200	C10A—H10B	0.9800
N8—H9N	0.9200	C10A—H10C	0.9800
N11—C12	1.481 (5)	C12—C13	1.531 (5)
N11—C10	1.485 (5)	C12—C12A	1.539 (5)
N11—H11N	0.8800	C12—H12	1.0000
C2—C3	1.524 (5)	C12A—H12A	0.9800
C2—H2A	0.9900	C12A—H12B	0.9800
C2—H2B	0.9900	C12A—H12C	0.9800
C3—C3A	1.518 (5)	C13—C14	1.533 (5)
C3—H3	1.0000	C13—H13A	0.9900
C3A—H3A1	0.9800	C13—H13B	0.9900
C3A—H3A2	0.9800	C14—C14B	1.526 (5)
C3A—H3A3	0.9800	C14—C14A	1.529 (5)
C5—C6	1.523 (5)	C14A—H14A	0.9800
C5—C5A	1.534 (5)	C14A—H14B	0.9800
C5—H5	1.0000	C14A—H14C	0.9800
C5A—H5A1	0.9800	C14B—H14D	0.9800
C5A—H5A2	0.9800	C14B—H14E	0.9800
C5A—H5A3	0.9800	C14B—H14F	0.9800
			
O4—Cl1—O3	109.1 (3)	C7B—C7—C6	110.4 (3)
O4—Cl1—O1	109.2 (3)	C7A—C7—C6	112.0 (3)
O3—Cl1—O1	108.9 (3)	N8—C7—C6	107.4 (3)
O4—Cl1—O2	111.1 (2)	C7—C7A—H7A1	109.5
O3—Cl1—O2	111.18 (19)	C7—C7A—H7A2	109.5
O1—Cl1—O2	107.27 (19)	H7A1—C7A—H7A2	109.5
O7—Cl2—O8	110.3 (2)	C7—C7A—H7A3	109.5
O7—Cl2—O5	110.3 (2)	H7A1—C7A—H7A3	109.5
O8—Cl2—O5	109.6 (2)	H7A2—C7A—H7A3	109.5
O7—Cl2—O6	109.1 (2)	C7—C7B—H7B1	109.5
O8—Cl2—O6	109.7 (2)	C7—C7B—H7B2	109.5
O5—Cl2—O6	107.8 (2)	H7B1—C7B—H7B2	109.5
C2—N1—C14	117.5 (3)	C7—C7B—H7B3	109.5
C2—N1—H1N	107.9	H7B1—C7B—H7B3	109.5
C14—N1—H1N	107.9	H7B2—C7B—H7B3	109.5
C2—N1—H2N	107.9	N8—C9—C10	112.2 (3)
C14—N1—H2N	107.9	N8—C9—H9A	109.2
H1N—N1—H2N	107.2	C10—C9—H9A	109.2
C5—N4—C3	114.7 (3)	N8—C9—H9B	109.2
C5—N4—H4N	109.0	C10—C9—H9B	109.2
C3—N4—H4N	107.0	H9A—C9—H9B	107.9
C9—N8—C7	118.3 (3)	N11—C10—C10A	116.3 (4)
C9—N8—H8N	107.7	N11—C10—C9	109.1 (3)
C7—N8—H8N	107.7	C10A—C10—C9	107.9 (4)
C9—N8—H9N	107.7	N11—C10—H10	107.7
C7—N8—H9N	107.7	C10A—C10—H10	107.7
H8N—N8—H9N	107.1	C9—C10—H10	107.7
C12—N11—C10	116.3 (3)	C10—C10A—H10A	109.5
C12—N11—H11N	109.5	C10—C10A—H10B	109.5
C10—N11—H11N	109.5	H10A—C10A—H10B	109.5
N1—C2—C3	111.9 (3)	C10—C10A—H10C	109.5
N1—C2—H2A	109.2	H10A—C10A—H10C	109.5
C3—C2—H2A	109.2	H10B—C10A—H10C	109.5
N1—C2—H2B	109.2	N11—C12—C13	109.9 (3)
C3—C2—H2B	109.2	N11—C12—C12A	114.5 (3)
H2A—C2—H2B	107.9	C13—C12—C12A	107.5 (3)
N4—C3—C3A	111.4 (3)	N11—C12—H12	108.3
N4—C3—C2	108.4 (3)	C13—C12—H12	108.3
C3A—C3—C2	112.7 (3)	C12A—C12—H12	108.3
N4—C3—H3	108.1	C12—C12A—H12A	109.5
C3A—C3—H3	108.1	C12—C12A—H12B	109.5
C2—C3—H3	108.1	H12A—C12A—H12B	109.5
C3—C3A—H3A1	109.5	C12—C12A—H12C	109.5
C3—C3A—H3A2	109.5	H12A—C12A—H12C	109.5
H3A1—C3A—H3A2	109.5	H12B—C12A—H12C	109.5
C3—C3A—H3A3	109.5	C12—C13—C14	117.7 (3)
H3A1—C3A—H3A3	109.5	C12—C13—H13A	107.9
H3A2—C3A—H3A3	109.5	C14—C13—H13A	107.9
N4—C5—C6	108.5 (3)	C12—C13—H13B	107.9
N4—C5—C5A	112.6 (3)	C14—C13—H13B	107.9
C6—C5—C5A	111.4 (3)	H13A—C13—H13B	107.2
N4—C5—H5	108.1	C14B—C14—N1	110.2 (3)
C6—C5—H5	108.1	C14B—C14—C14A	110.4 (3)
C5A—C5—H5	108.1	N1—C14—C14A	105.1 (3)
C5—C5A—H5A1	109.5	C14B—C14—C13	109.7 (3)
C5—C5A—H5A2	109.5	N1—C14—C13	108.5 (3)
H5A1—C5A—H5A2	109.5	C14A—C14—C13	112.8 (3)
C5—C5A—H5A3	109.5	C14—C14A—H14A	109.5
H5A1—C5A—H5A3	109.5	C14—C14A—H14B	109.5
H5A2—C5A—H5A3	109.5	H14A—C14A—H14B	109.5
C5—C6—C7	116.9 (3)	C14—C14A—H14C	109.5
C5—C6—H6A	108.1	H14A—C14A—H14C	109.5
C7—C6—H6A	108.1	H14B—C14A—H14C	109.5
C5—C6—H6B	108.1	C14—C14B—H14D	109.5
C7—C6—H6B	108.1	C14—C14B—H14E	109.5
H6A—C6—H6B	107.3	H14D—C14B—H14E	109.5
C7B—C7—C7A	110.3 (3)	C14—C14B—H14F	109.5
C7B—C7—N8	110.5 (3)	H14D—C14B—H14F	109.5
C7A—C7—N8	106.0 (3)	H14E—C14B—H14F	109.5
			
C14—N1—C2—C3	180.0 (3)	C7—N8—C9—C10	−179.3 (3)
C5—N4—C3—C3A	−63.2 (4)	C12—N11—C10—C10A	49.5 (5)
C5—N4—C3—C2	172.2 (3)	C12—N11—C10—C9	171.8 (3)
N1—C2—C3—N4	62.0 (4)	N8—C9—C10—N11	70.5 (4)
N1—C2—C3—C3A	−61.8 (4)	N8—C9—C10—C10A	−162.3 (4)
C3—N4—C5—C6	169.6 (3)	C10—N11—C12—C13	152.2 (3)
C3—N4—C5—C5A	−66.6 (4)	C10—N11—C12—C12A	−86.7 (4)
N4—C5—C6—C7	−63.7 (4)	N11—C12—C13—C14	−61.2 (4)
C5A—C5—C6—C7	171.8 (3)	C12A—C12—C13—C14	173.6 (3)
C9—N8—C7—C7B	−37.0 (4)	C2—N1—C14—C14B	−44.5 (4)
C9—N8—C7—C7A	−156.5 (3)	C2—N1—C14—C14A	−163.4 (3)
C9—N8—C7—C6	83.6 (4)	C2—N1—C14—C13	75.7 (4)
C5—C6—C7—C7B	−173.8 (3)	C12—C13—C14—C14B	−171.8 (3)
C5—C6—C7—C7A	−50.5 (4)	C12—C13—C14—N1	67.7 (4)
C5—C6—C7—N8	65.6 (4)	C12—C13—C14—C14A	−48.3 (4)

### Hydrogen-bond geometry (Å, °)

**Table:**

*D*—H···*A*	*D*—H	H···*A*	*D*···*A*	*D*—H···*A*
N1—H2n···N11	0.92	2.07	2.841 (4)	140
N8—H9n···N4	0.92	1.95	2.763 (5)	146
N1—H1n···O2	0.92	2.37	2.963 (4)	122
N1—H1n···O7^i^	0.92	2.41	3.169 (4)	140
N1—H2n···N4	0.92	2.56	2.929 (4)	105
N4—H4n···O6	0.88	2.38	3.212 (4)	158
N8—H8n···O1	0.92	2.02	2.937 (5)	177
N11—H11n···O6	0.88	2.24	3.087 (5)	162
C2—H2a···O1^ii^	0.99	2.57	3.109 (5)	114
C9—H9a···O5^iii^	0.99	2.57	3.498 (6)	155
C12a—H12b···O8	0.98	2.56	3.487 (5)	158

Symmetry codes:  (i) *x*, −*y*+1/2, *z*+1/2; (ii) *x*+1, *y*, *z*; (iii) *x*−1, *y*, *z*.
